# Assessing training needs and influencing factors among personnel at centers for disease control and prevention in northeast China: a cross-sectional study framed by SDT and TPB using machine learning techniques

**DOI:** 10.1186/s12889-025-23393-w

**Published:** 2025-06-10

**Authors:** Kexin Wang, Peng Wang, Min Wei, Yanping Wang, Huan Liu, Ruiqian Zhuge, Qunkai Wang, Nan Meng, Yiran Gao, Yuxuan Wang, Lijun Gao, Jingjing Liu, Xin Zhang, Mingli Jiao, Qunhong Wu

**Affiliations:** 1 Department of Epidemiology, School of Public Health, Harbin Medical University, Harbin, Heilongjiang Province China; 2 Department of Social Medicine, School of Health Management, Harbin Medical University, Harbin, Heilongjiang Province China; 3 Department of Health Policy, School of Health Management, Harbin Medical University, Harbin, Heilongjiang Province China; 4 School of Public Health, Anhui University of Science and Technology, Hefei, Anhui Province China; 5 Joint Research Center of Occupational Medicine and Health, Institute of Grand Health, Hefei Comprehensive National Science Center, Anhui University of Science and Technology, Hefei, Anhui Province China; 6 Department of Human Resources, School of Health Management, Harbin Medical University, Harbin, Heilongjiang Province China

**Keywords:** Training needs, Public health personnel, Competency, Latent class analysis, Machine learning, Boruta algorithm, Theory of planned Behavior, Self-Determination Theory

## Abstract

**Objectives:**

Training public health personnel is crucial for enhancing the capacity of public health systems. However, existing research often falls short in providing a comprehensive theoretical framework and fails to account for the intricate interplay of multi-dimensional factors in public health. This study aims to identify knowledge and skill gaps at both individual and organizational levels, and to explore multi-dimensional factors influencing training needs within the theoretical frameworks of the Theory of Planned Behavior and Self-Determination Theory.

**Methods:**

This cross-sectional study used stratified cluster sampling to conduct an online survey among personnel at the Centers for Disease Control and Prevention from Heilongjiang Province, Jilin Province, Liaoning Province, and Inner Mongolia Autonomous Regions during May 2023. A total of 11,912 valid questionnaires were collected. Latent Class Analysis was used to analyze competency subgroups covering professional abilities, general abilities, and management abilities. Boruta algorithm was used to select feature and improve the performance of the following predictive models. Logistic regression, random forest, least absolute shrinkage and selection operator (LASSO), and extreme gradient boosting (XGBoost) were used to predict training needs and explore the impact of various multi-dimensional factors. SHapley Additive exPlanations (SHAP) were used to explain the output of the optimal machine learning model.

**Results:**

This study identified the four subgroups of competency patterns, including novice (25.3%), public health experts (15.1%), potential expansion talents (24.7%), and versatile talents (34.9%). Boruta algorithm identified 9 confirmed variables, 3 tentative variables, and 30 rejected variables. Compared with other models, XGBoost model demonstrated the best performance. The value of AUC was 0.702, and the value of accuracy, precision, recall, and F1 score was 0.6485, 0.6564, 0.6301, and 0.6430, respectively. The SHAP based on XGBoost model indicated on-job training satisfaction had a strong association with training needs among public health personnel. Self-improvement needs, college education satisfaction, workload, competency patterns, and team cohesion were also important factors.

**Conclusions:**

Intrinsic motivation is the key factor influencing the training needs of public health personnel. When formulating training plans, priority should be given to how to improve on-job training satisfaction and design a more targeted competency patterns tailored training curriculum. Moreover, organizational incentives aimed at motivating trainees and integrating career development goals into training program design are important. Therefore, setting training priorities becomes key to help ensure that training programs are targeted and effective, thereby promoting individual and organizational career development.

**Supplementary Information:**

The online version contains supplementary material available at 10.1186/s12889-025-23393-w.

## Introduction

The effectiveness of public health largely depends on the competence and training of professionals. In the face of emerging infectious diseases, chronic conditions, and other public health challenges, enhancing the professionalism of public health personnel is especially crucial. Therefore, exploring influencing factors of training needs is essential for optimizing training strategies to make public health personnel possess a diverse set of skills and adapt to complex health environments and evolving social needs.

Previous studies demonstrate that the training needs are influenced by various factors, including an individual’s educational background [1], working environment [2, 3], social support [4], and competency [5, 6], yet lacking a solid theoretical foundation to integrate multi-dimensional factors and analyze the interactions. A study in Heilongjiang Province employs logistic regression to explore the influencing factors of coping capacity among Centers for Disease Control and Prevention (CDC) personnel [7]. Some studies provide a cross-sectional description of training conditions for rural doctors in China, detailing training duration, methods, and contents; however, they lack a deep analysis of training needs [8]. Adult learning theory is utilized to emphasize learners’ self-directed abilities, and organizational behavior theory to investigate the impact of organizational support on training needs in some studies. For example, intrinsic motivations, such as personal growth and the need to stay updated with advancements in public health practice, are highlighted to be driving factors of seeking training [9]. However, these studies often adopt a single theoretical perspective, failing to fully consider the multi-dimensional factors influencing training needs. Comparable reflections on how workforce competences should be aligned with targeted training in rapidly changing organizational settings have also been advanced in Europe [10]. Therefore, this study aims to integrate the Theory of Planned Behavior (TPB) and Self-Determination Theory (SDT) to establish a more comprehensive analytical framework to uncover the complex dynamics of training needs among public health personnel and to enhance our understanding of the deeper motivations behind their behaviors.

This study uses feature selection and machine learning methods to uncover the underlying multi-factor mechanisms. Feature selection can effectively identify the variables that most influence training needs, enhancing both the accuracy and interpretability of the model. By comparing various machine learning algorithms, such as logistic regression (LR), random forest (RF), least absolute shrinkage and selection operator (LASSO), and extreme gradient boosting (XGBoost), we can comprehensively evaluate the performance of each algorithm in predicting training needs. This comparison not only highlights the relative importance of each factor but also provides a more reliable foundation for developing and implementing public health education policies, ultimately promoting the enhancement of training strategies.

Access to effective education and training for public health personnel is limited, with only 20% of the U.S. personnel receiving the most effective education and training [11]. Prioritizing the allocation of limited training resources to the public health personnel with clearly identified training needs is beneficial for improving the return on training investment and maximizing employee performance [12]. Therefore, assessing the personnel training needs is crucial for determining the priority order for public health training and providing effective training programs [13]. This study aims to identify knowledge and skill gaps at both the individual and sector levels; to explore influencing factors of training needs from individual, organizational, and socio-environmental levels by integrating SDT with TPB; and to determine which individuals should receive training to enhance the impact of training initiatives and improve overall training effectiveness.

## Methods

### Theoretical framework

**Fig. 1:**
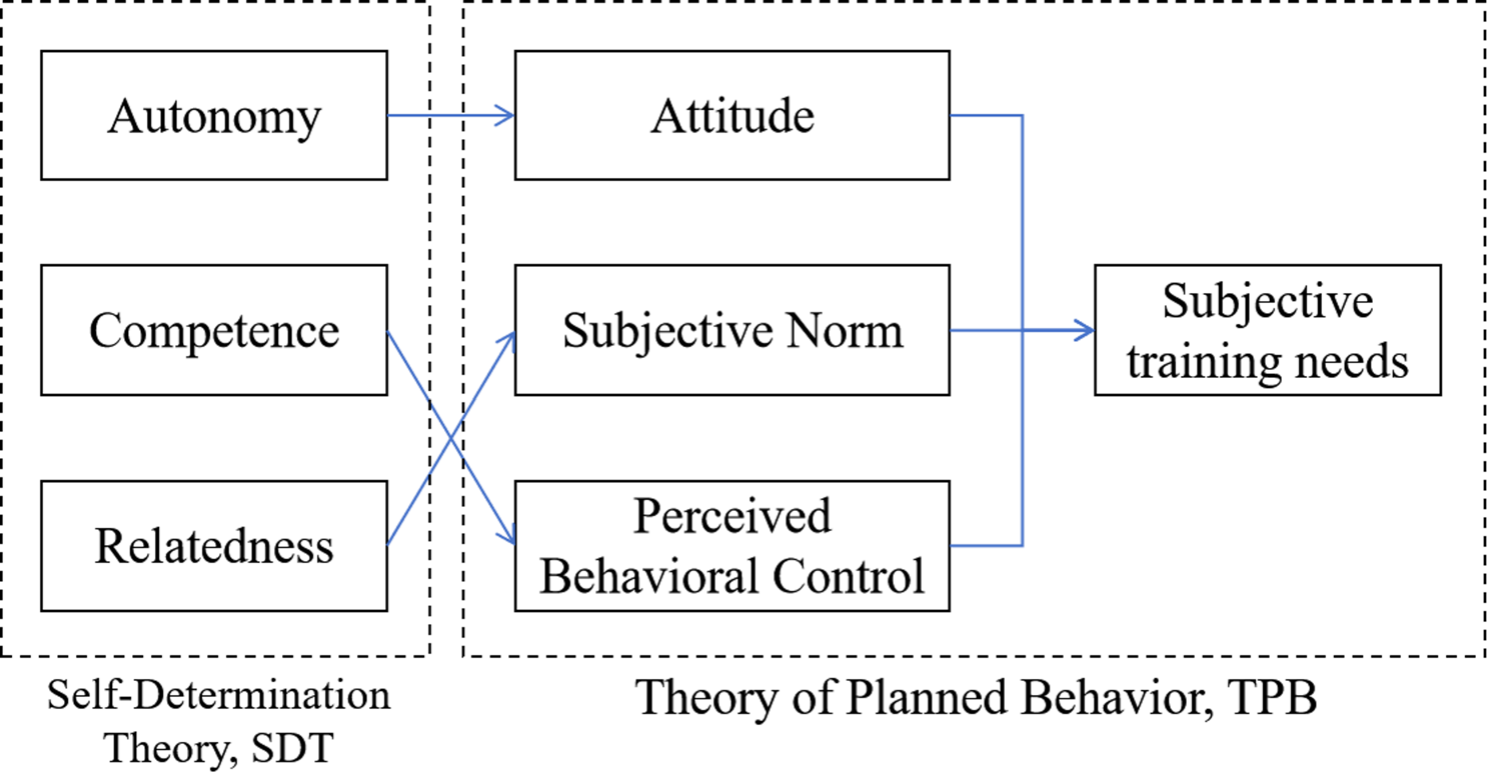
The theoretical framework of this study

This study integrates the TPB and SDT to analyze the interactions of individual, organizational, and socio-environmental factors on the training needs of public health personnel (As shown in Fig. 1). TPB encompasses attitudes, subjective norms, and perceived behavioral control, analyzing the external environment’s impact on training needs. Attitudes reflect perceptions of training value, while subjective norms indicate the influence of others’ expectations. Perceived behavioral control assesses perceived barriers and resource availability that affect training participation. SDT focuses on internal motivation and psychological needs, specifically autonomy, competence, and relatedness. Autonomy affects an individual’s willingness and attitude toward training. Competence boosts confidence in participating and shapes expectations about training outcomes. Relatedness addresses the need for emotional and social support from the organization, which influences subjective norms.

The combination of TPB and SDT can integrate multiple factors and enhance the explanatory power. The combination of individual intrinsic motivation and external environment provides a more comprehensive perspective for analyzing training needs. Compared with the traditional single-theory research, the integrated theoretical framework can more accurately capture the formation mechanism of training needs. Theoretical frameworks are also able to capture the interaction of intrinsic and extrinsic motivations. SDT focuses on intrinsic motivation, while TPB focuses on external behavioral control. Integrating the two provides a deeper understanding of how an individual’s intrinsic motivation in choosing to receive training is modulated by external factors. For example, an individual’s sense of competence may be influenced by organizational emotional and external support, which affects their training needs.

### Study design and data collection

This cross-sectional study used stratified cluster sampling to conduct an online survey towards CDC personnel from Heilongjiang Province, Jilin Province, Liaoning Province, and Inner Mongolia Autonomous Region in May 2023. A total of 11,912 valid questionnaires was collected with an effective response rate of 98.04% [14]. Participants were excluded: (1) 67 participants who didn’t agree to answer the questionnaire; (2) 109 participants who selected the option “Please do not choose this choice” in a particular question; (3) 62 participants who answered the questionnaire with logic errors, such as work year > age. The reliability and validity of the questionnaire were excellent, with Cronbach’s coefficient of 0.962 and KMO value of 0.960.

### Ethical considerations

Ethical approval for this study was obtained from the ethics review board of Harbin Medical University, and all participants provided informed consent before participating in the survey.

### Variables

This study adopted self-designed questionnaire to investigate the sociodemographic information, work status, self-competency satisfaction, education satisfaction, training satisfaction, training purposes, job satisfaction, social supports, funds support, training weakness, trainees’ subjective issues, and training needs. The dependent variable training needs was scored from 1 (Not need at all) to 5 (Strongly need). In this study, a score ranging from 1 to 3 indicated “Low needs”, assigned a value of 0, while a score ranging from 4 to 5 indicated “High needs”, assigned a value of 1. Participants rated 24 items related to public health competency on a scale of 1 to 5, with higher score indicating highly satisfaction. A score ranging from 1 to 3 indicated “Low”, assigned a value of 0, while a score ranging from 4 to 5 indicated “High”, assigned a value of 1. The details of variables were provided in Table S1.

### Statistical analysis

Categorical variables were described with frequency and percentage. Chi-Square test was used to assess the associations between training needs and other independent variables. Latent class analysis (LCA) was used to identify the competency patterns. Feature selection and machine learning models were used to explore influencing factors of training needs. Furthermore, sensitivity analysis was conducted to evaluate the stability of the model.

### Latent class analysis

Latent class analysis can identify latent subgroups and explain group heterogeneity based on individual response characteristics to a set of categorical observation indicators [15]. We used LCA to identify competency patterns through 24 items related to public health abilities. Model fit index was adopted to select the optimal model, including Akaike Information Criterion (AIC), Bayesian Information Criterion (BIC), Sample-Size Adjusted Bayesian Information Criterion (SSA-BIC), Lo-Mendell-Rubin likelihood ratio test (LMR-LRT), Bootstrapped Likelihood Ratio Test (BLRT), and Entropy [16]. Smaller AIC, BIC, and SSA-BIC indicated better fit [17, 18]. Significant LMR-LRT and BLRT values indicated that the k class model was a better fit than the k-1 class model [19]. The precision of classification models was evaluated using relative entropy values closer to 1.00, indicating good classification for participants in the sample. MPlus (Version 8.3) was used to analyze LCA.

### Feature selection

Feature selection was performed using the Boruta algorithm, which identified features associated with the response variable by comparing Z-values with those of shadow features created through random forest voting [20]. If the Z-value of a real feature surpassed the maximal Z-value of shadow features in multiple independent trials, it was considered “important”. Features deemed highly unimportant were constantly removed. The Boruta algorithm stopped when all features were either confirmed or rejected, or when it reached a specified limit of random forest. This process identified features strongly or weakly relevant to the response variable. The complete data was split into training set (70% randomly selected samples) and test set (the remaining 30% sample). The train set was used to analyze feature selection.

### Machine learning models

Four machine learning algorithms, including LR, RF, LASSO, and XGBoost, were employed. In the training set, hyperparameter optimization and ten-fold cross-validation were leveraged to optimize parameters of four predictive models. The logistic regression model was developed as a reference. Youden Index was utilized to balance the relationship between the expected value and volatility of the predicted variable within the training set, and to enable the selection of an optimal threshold value (cutoff point) [21]. In the test set, accuracy, precision, recall, F1 score, and the area under the receiver operating characteristic curve (AUC) were calculated to evaluate the performance of each predictive model [22] and the 95% confidence intervals (CIs) were also reported using the bootstrap method and 1,000 bootstrap iterations for all metrics. SHapley Additive exPlanations (SHAP) were used to explain the output of the optimal machine learning model, providing a quantitative analysis of the importance and contribution of features to the model’s predictions [23]. Both feature selection and machine learning were conducted by using R 4.3.2 and a two-sided *P* <.05 was regarded as statistically significant.

## Results

### Competency patterns for the latent class analysis

**Fig. 2:**
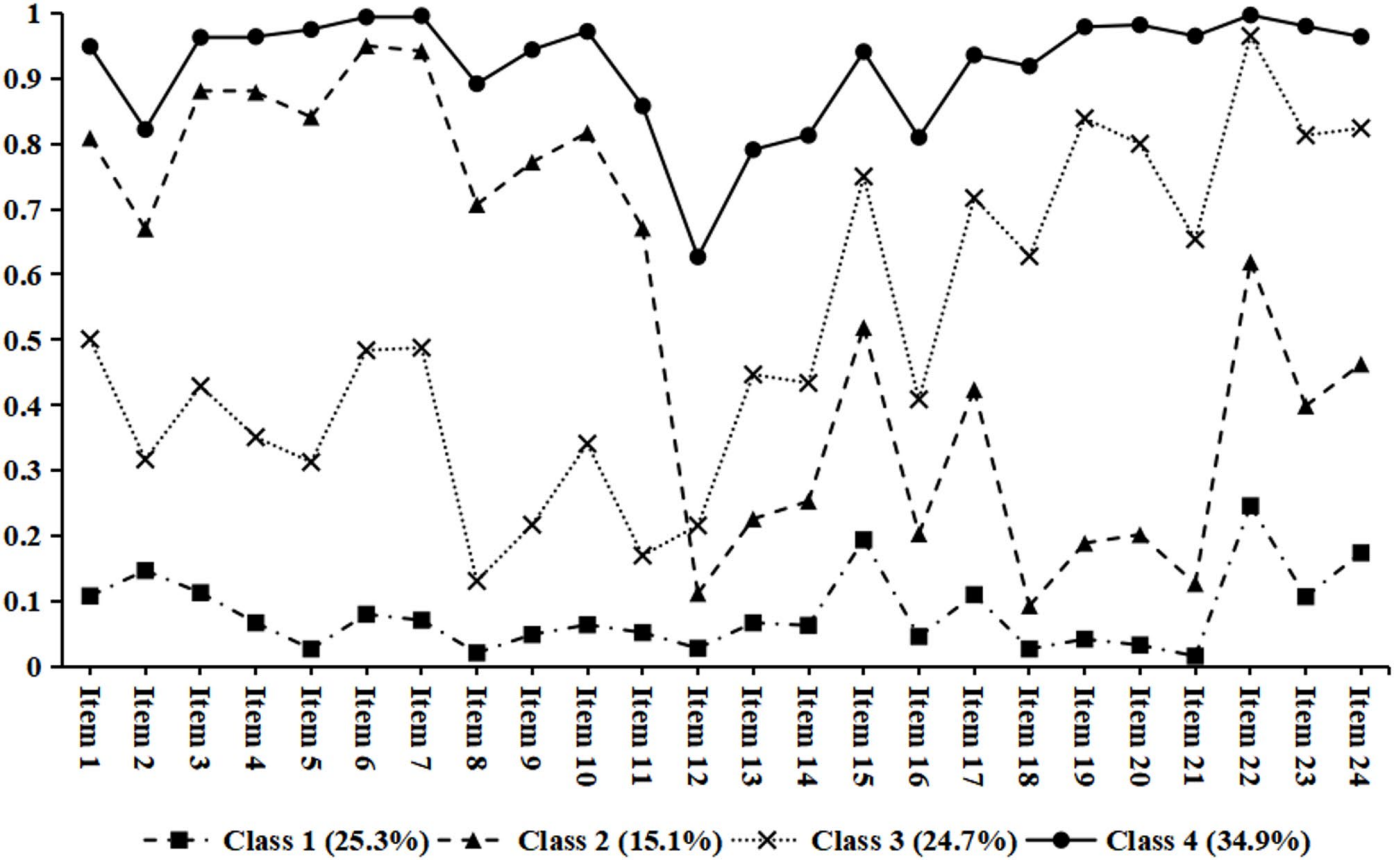
The competency patterns among CDC personnel by using the latent class model

Table S2 showed the fitting index of the latent class analysis, indicating Model 4 was the optimal latent class model. Figure 2 and Table S3 presented the estimated item probabilities for the four identified latent class. Class 1 (*n* = 3008, 25.3%) was labeled as “Novice”, because participants in this class reported the lowest proficiency with all the items. Class 2 (*n* = 1798, 15.1%) was labeled as “Public health experts”, who were satisfied with public health professional abilities but were not proficient in general abilities. Class 3 (*n* = 2944, 24.7%) was labeled as “Potential expansion talents”, who were more proficient in general abilities but not public health professional abilities. Class 4 (*n* = 4162, 34.9%) was labeled as “Versatile talents”, because its members excelled in all aspects.

Figure S1 showed the average score of the items in the four different competency patterns. Versatile talents (Class 4) showed excellence (average score greater than 4) in most of the competency items, such as team collaboration ability (Item 22, 4.35), field investigation and control capability (Item 7, 4.32) and emergency response and management capability (Item 6, 4.31), but below 4 score in scientific research capability (Item 13, 3.71), document writing ability (Item 12, 3.97) and data analysis capability (Item 14, 3.99). Public health experts (Class 2) excelled in emergency response and management capability (Item 6, 4.11), as well as field investigation and control capability (Item 7, 4.10), but showed less proficiency in scientific research capability (Item 13, 2.83) and leadership decision making capability (Item 18, 2.97). Potential expansion talents (Class 3) mastered the team collaboration ability (Item 22, 4.12), while lacked experience in community diagnosis and care capability (Item 8, 2.91), basic clinical skills (Item 11, 2.91), as well as scientific research capability (Item 13, 2.95). The details of distribution of competency patterns across demographic and job-related characteristics were shown in Figure S2.

### Participants’ characteristics

Table S4 summarized the characteristics of the 11,912 participants. Half of the participants reported high training needs (2,985, 50.2%). There was a higher proportion of females (8,036, 67.5%) compared to males (3,876, 32.5%). The majority of participants were aged 31–40 years (3,332, 28.0%), held a Bachelor’s degree (7,400, 62.1%), worked in Inner Mongolia Autonomous Region (4,610, 38.7%), and were employed at the county/district-level CDC (7,853, 65.9%). All of these characteristics were significantly associated with training needs (*P* <.05).

### Feature selection

**Fig. 3:**
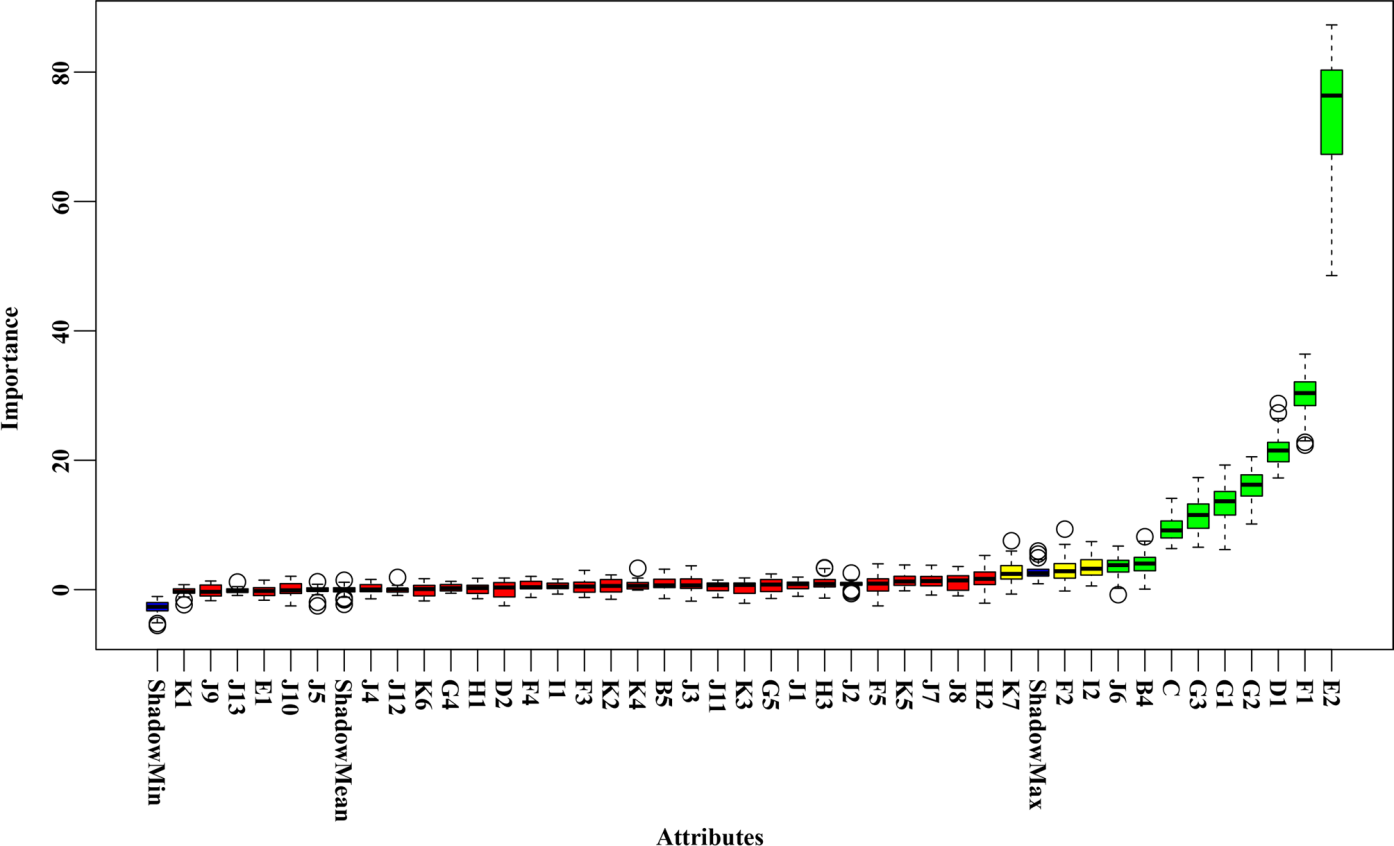
Feature selection based on the Boruta algorithm

Feature selection based on the Boruta algorithm identified 9 confirmed variables, 3 tentative variables, and 30 rejected variables in terms of Z-value, as shown in Fig. 3.

### Model performance comparisons

**Fig. 4:**
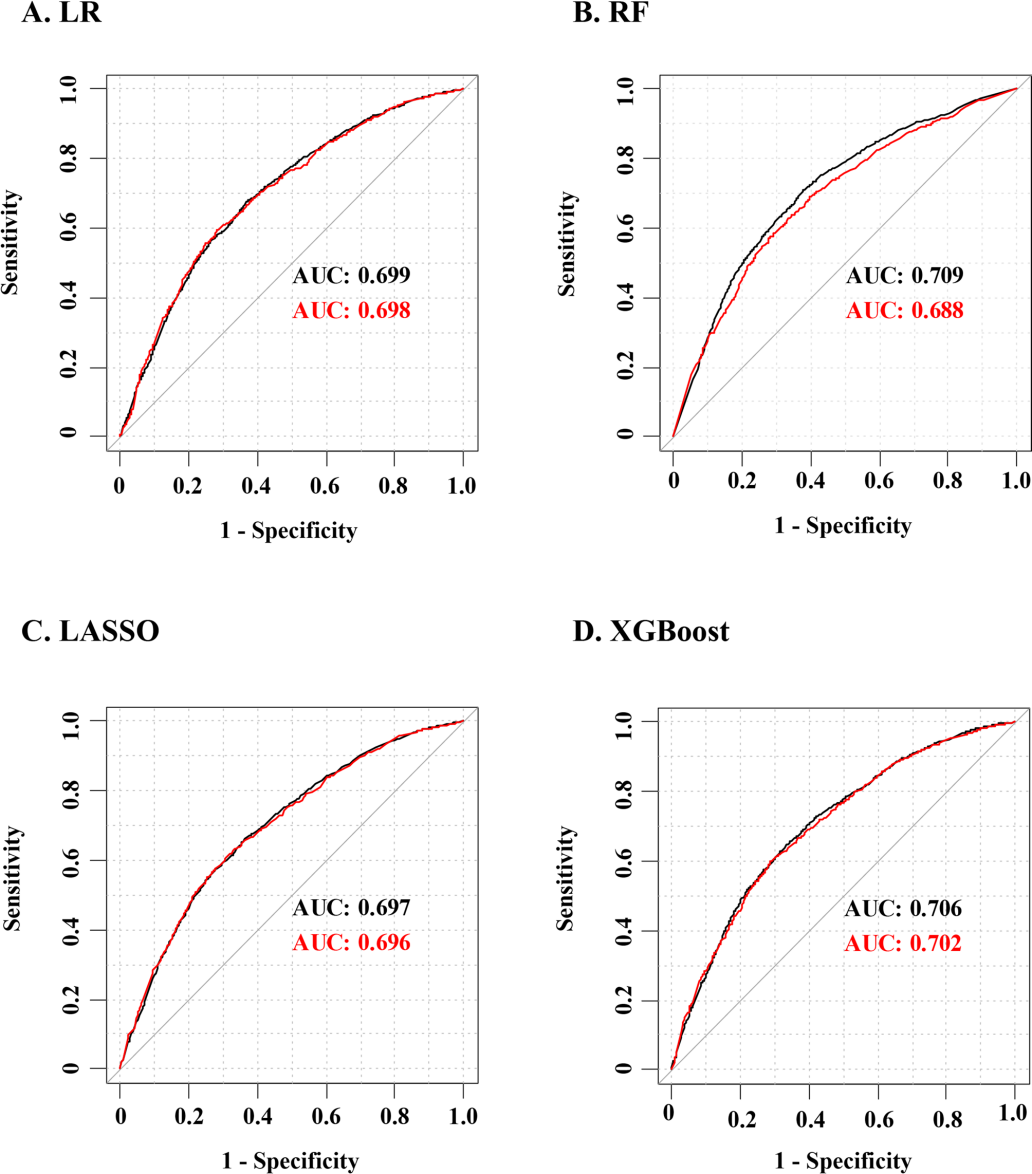
Comparison of ROC curves and AUC for four machine learning models. (black line, train set; red line, test set)

The AUC values were listed to evaluate the classification effectiveness of the four ML models (Fig. 4). In general, the difference of AUC values between the training set and the test set was small, which indicated that the prediction effect of the models on different data sets was more consistent. The AUC of logistic regression, LASSO and XGBoost on the training set and the test set were very close, indicating that the model has good generalization ability, while the test set AUC of random forest was slightly lower than that of the training set, showing a slight overfitting phenomenon. Considering the model performance metrics (Fig. 4, Table S5, and Figure S3), the XGBoost model stands out as the optimal choice due to its strong performance on the test set, along with its robust stability and generalization capabilities.

### Feature importance in XGBoost model

**Fig. 5:**
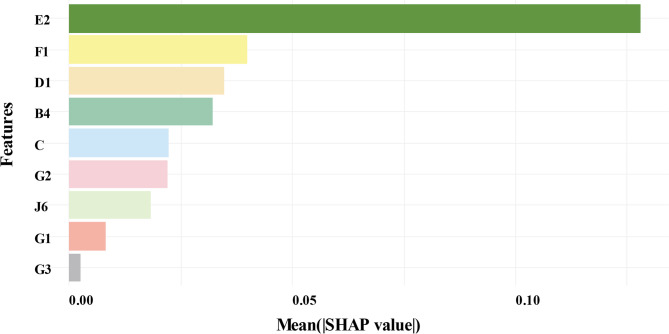
Feature importance in XGBoost model predictions based on mean SHAP values

Figure 5 showed the average SHAP values for different features to measure the importance of each feature to the XGBoost model output. The on-job training satisfaction showed the highest average SHAP value, indicating a strong association with training needs among public health personnel. Self-improvement needs, college education satisfaction, and workload were also prominently associated, as reflected by their higher SHAP values. Competency patterns and team cohesion, while demonstrating lower average SHAP values, still exhibited measurable associations.

### Sensitivity analysis

Table S6 reflected the performance of four machine learning models grouped by sex, age, education level, and nationally-certified professional title. Among these models, the XGBoost algorithm demonstrated the best overall performance, achieving the highest AUC values across almost all demographic groups. Additionally, it exhibited a high degree of stability, consistently yielding reliable predictions regardless of the variations in the grouped parameters. This stability indicated that the XGBoost model was robust in its ability to generalize across different subsets of the population, making it a preferred choice for further analysis and application.

## Discussion

### Principal findings

This study utilizes a combined SDT and TPB theoretical framework to comprehensively examine the interaction between individual intrinsic motivation and external environmental factors, offering a fresh perspective on the training needs of public health personnel. One of our primary findings is to identify knowledge and skill gaps that public health personnel generally lack scientific research ability. Public health experts are better at emergency response and management capability, field investigation and control capability, epidemiological tracing capability, as well as surveillance and early warning capability after the experience of COVID-19. Additionally, in the Centers for Disease Control and Prevention, a functional organization with high standards for public health professional competence, the proportion of public health experts remains relatively small. Another key finding is that intrinsic motivation are strongly associated with training needs among public health personnel, rather than external circumstances.

### Skill gaps in public health personnel with different competency patterns

Public health versatile talents and public health experts comprise 50.0% of the total CDC personnel. They excel in field investigation and control, emergency response and management, as well as epidemiological tracking. This may be attributed to China’s dynamic zero-COVID policy, which employs effective measures such as large-scale testing and isolation to precisely manage localized outbreaks [24]. This policy sets high standards for the competencies of public health personnel, who must leverage advanced technologies like big data and artificial intelligence to quickly identify close contacts and promptly interrupt transmission chains, as well as to detect and predict pandemic trends [25]. In addition, multiple training sessions and practical experience during the COVID-19 period have helped public health personnel greatly improve their professional capabilities.

Almost all public health personnel generally exhibit deficiencies in scientific research capability and data analysis capability. Education might be the most direct factor, as 91.2% of participants held bachelor’s degree or below in our study. Northeastern economic development in China lags behind other regions, which may lead to a scarcity of resources, including funds, equipment, and training opportunities, thus restricting the development of scientific research activities. Furthermore, the Northeastern CDC may not acknowledge or value the importance of scientific research and its outcomes. In fact, scientific research and data analysis are the primary core competencies of public health professionals [26]. Their value lies in helping public health workers understand the burden of disease, disability, and injury, providing opportunities to improve health across the life course, inferring causal mechanisms, and offering evidence for the impact of interventions [27]. Public health professionals urgently need to enhance their data science capabilities, as multimodal data and advanced analytical methods can bridge gaps in existing public health research and intervention practices [28, 29]. Moreover, the inclusion of scientific research ability in performance appraisals may stimulate enthusiasm and motivation among public health workers to engage in scientific research. In addition, we could nurture public health professionals with strategic scientific capabilities by fostering collaborations between medical universities and the CDC [30].

### The chain reaction of autonomy and attitude in influencing training needs

On-job training satisfaction was pivotal for determining training needs, which reflected trainees’ feelings about the entire training activity [31], such as the effectiveness of training content, the appropriateness of training methods, and the professionalism of trainers. Previous research demonstrated a positive relationship between job training satisfaction and training needs, which was consistent with our study. When trainees were satisfied with past training, they were more inclined to believe that they have acquired valuable knowledge and skills. Consequently, they exhibited higher motivation for learning and a greater willingness to participate in future training sessions, making the need for further training more pressing [32]. In our study, participants reported the problems and issues regarding on-job training satisfaction, for example, the lack of practical skills training (42.1%), lack of standardized training bases (41.6%), training content cannot be set as needed (41.4%), lack of advanced curriculum and teaching methods (34.6%), lack of advanced teaching and scientific research experimental equipment (34.0%), lack of a systematic training assessment system (32.5%), insufficient informationization construction (32.1%), training is just a formality (31.9%), the short training time and didn’t get it (31.0%), and training content is not highly relevant to the position (30.7%). Evidence from Italy demonstrated that trainees’ perception of the efficiency, usefulness of the training, and trainer performance were the influencing factors for training satisfaction [33]. Therefore, emphasizing the significance of each training session for public health personnel and enhancing training satisfaction can effectively elevate their willingness and demand for subsequent training opportunities.

This study also highlighted the importance of aligning training needs with career development goals, particularly focusing on self-improvement. Employees may require training to enhance specific skills either to fulfill the demands of their current roles or to prepare themselves for future career progression. In this study, 91.5% of participants expressing self-improvement needs reported high training needs, while 51.8% of those with job-improvement needs indicated high training needs. Some evidence suggested that not only did training have an effect on career development [34], but also that career development could improve basic skills, abilities, knowledge and attitudes through training, further promoteing organizational development [35]. For public health personnel seeking self-improvement, their career development aligned with the organization’s goals and met organizational needs. These public health workers took the lead in seeking training opportunities, proactively acquiring knowledge and skills, and consistently progressing. Therefore, prioritizing training for those with clear career development plans and goals not only enhances the overall quality and performance of employees but also fosters talent development and facilitates industry advancement.

### Competence-based intrinsic motivation emerges strong correlation with high training needs

This study also discovered that public health workers with heavy workload (2755, 53.7%) or public health versatile talents (2389, 57.4%) had higher training needs. It was consistent with the findings of another study that examined the associations between competencies and willingness to undergo training in public health among non-managers, middle managers, and upper managers [36]. In this study, public health experts and potential expansion talents reported lower training needs, considering the reasons of “conflict of working time and studying time” (48.5%) and “multiple responsibilities in one position, no time to study” (45.8%). Therefore, the competeny-based training was recommended, which broke down jobs into specific competencies or skills and then used those competencies to create a training program that could meet individual needs and learning styles. The Michigan Center for Public Health Preparedness developed competency-based preparedness training and evaluated the impact of knowledge-centered training activities on the development of performance competencies [37]. A study from Kansas also evaluated the effectiveness of a pilot competency-based public health training program [38]. When developing and delivering training, it’s crucial to evaluate the interests of your target audience, public health trainees [36]. This involves identifying their overall requirements, preferred training methods, and specific needs based on different audience types through a comprehensive training assessment process [39]. Therefore, it is recommended to carry out competency-based training, which can not only meet the needs of improving abilities, but also improve training satisfaction and further provide positive feedback on training needs.

### Strengths and limitations

Generally, grounding our investigation in the integrated framework of Self-Determination Theory (SDT) and Theory of Planned Behavior (TPB), this study explores the impact of intrinsic motivation and the external environment on the training needs of public health personnel. This dual-theory approach provides a comprehensive perspective, aiding in the identification of key factors influencing training needs. Additionally, we investigate the innovative use of feature selection and various machine learning methods to reduce noise in traditional logistic regression models, enhancing their flexibility and accuracy. This approach not only improves the ability to capture complex data relationships but also increases the model’s applicability across different contexts. Finally, by employing SHAP, this study enhances the interpretability of the machine learning model, making the outputs easier to understand and interpret. This transparency is crucial for policymakers to grasp the model’s predictions in practical applications, thereby further driving empirical research and policy development in the public health field.

However, several limitations must be considered when interpreting the results from this study. First, the results are based on CDC workers from the four provinces northeast of China, and may not represent the views of all the public health personnel. Second, training needs is a measurable gap between “what currently is” and “what should be”, the definition of which should be conducted by a complete assessment process [40]. However, training needs for this study were measured in the form of self-reports. Although self-report provided a valid measure, it may be affected by participants’ personal biases and misunderstandings. Therefore, these factors need to be taken into account carefully when interpreting the findings, and results from other assessment methods need to be considered as a reference to ensure a comprehensive understanding of training needs. Third, while models utilizing feature selection and machine learning methods demonstrate better performance than traditional logistic regression, they still fall short compared to clinical predictive models overall. Possible reasons for this gap include the reliance of clinical predictive models on extensive domain expertise and experience, which allows them to combine multiple clinical features and indicators to more accurately capture disease progression and patient status. Although advanced algorithms are employed in this study, the complexity and nuances of real-world scenarios may not be fully addressed during feature selection and data processing. Additionally, while the low correlation and multicollinearity among the variables in this study suggest greater independence of the features, this may also result in machine learning performance being similar to that of traditional logistic regression. Low multicollinearity can limit the ability of machine learning models to capture nonlinear relationships and complex interaction effects, thereby impacting their overall predictive performance. However, it is important to note that the advantages of machine learning models are likely to be more pronounced when applied to other datasets, particularly those exhibiting multicollinearity. Fourth, the flexibility and adaptability of machine learning models across different datasets will provide robust support for future research, however, we acknowledge that statistically significant variables may not always translate to practically important factors, and vice versa. This indicates that future work should focus on validating machine learning approaches in more complex and diverse data environments to better inform decision-making and practice in public health.

## Electronic supplementary material

Below is the link to the electronic supplementary material.

Supplementary Material 1

## Data Availability

The raw data supporting the conclusions of this article will be made available by the corresponding authors.
