# Supplementary material for: Assessing training needs and influencing factors among personnel at centers for disease control and prevention in northeast China: a cross-sectional study framed by SDT and TPB using machine learning techniques
Source: BMC Public Health. 2025 Jun 10;25:2157. doi: 10.1186/s12889-025-23393-w (PMC12150469; doi:10.1186/s12889-025-23393-w)

Table S1. The details (code, full name, questionnaire wording, and response options) of variables.

| Code | Full variable name | Questionnaire wording | Response options and levels |
| --- | --- | --- | --- |
| Not applicable | Training needs | Please assess the degree of your training needs by yourself. | Likert 5, from 1 (not need at all) to 5 (strongly need).  The score ranging from 1 to 3 indicated “Low needs”, with the value of “0”; while the score above 3 indicated “High needs”, marked as “1”. |
| A1 | Sex | Sex: (1) Male (2) Female | Male = 1; Female = 2 |
| A2 | Age | Age: ____ years old | ≤ 30 = 1; 31-40 = 2; 41-50 = 3; ≥ 51 = 4; |
| A3 | Educational level | Educational background：(1) High school diploma or below (2) Associate degree (3) Bachelor’s degree (4) Master’s degree (5) Doctoral degree | College degree or below = 1;  Bachelor's degree = 2;  Above bachelor's degree = 3; |
| A4 | Nationally-certified Professional Title (Job title) | Nationally-certified Professional Title: (1) Senior (2) Associate senior (3) Intermediate/Lecturer-level position (4) Junior | Junior = 1; Intermediate = 2; Senior = 3 |
| A5 | Region | Work location: ____Province/Municipality/Autonomous Region ____City ____County/District | Heilongjiang Province = 1; Jilin Province = 2; Liaoning Province = 3; Inner Mongolia Mongolia Autonomous = 4; |
| A6 | Institutional classification | Institutional classification: (1) National level (2) Provincial/municipal level (3) Prefectural level (4) County/District-level | Provincial or municipal level = 1; Prefectural level = 2; County or district level = 3; |
| A7 | Income monthly (RMB) | Personal income monthly (RMB): (1) below 3000 (2) 3000-5000 (3) 5001-7000 (4) 7001-8000 (5) above 8000 | below 3000 = 1; 3000-5000 = 2; 5001-7000 = 3; 7001-8000 = 4; above 8000 = 5; |
| B1 | Administrative department | Work department [multiple choices]: (1) Administrative department (2) Chronic non-communicable disease prevention and control (3) Infectious disease prevention and control (4) Sexually transmitted disease and AIDS prevention and control (5) Mental illness prevention and treatment (6) Viral disease prevention and control (7) Skin disease prevention and treatment (8) Tuberculosis prevention and control (9) Endemic disease prevention and control (10) Occupational health (11) Environmental health (12) Nutrition and food hygiene safety (13) School health (14) Radiation and nuclear safety protection (15) Toxicology and pathology testing (16) Aging health (17) Disinfection and hospital infection control (18) Public health supervision (19) Medical health supervision (20) Disease surveillance and information management (21) Emergency response to public health emergencies (22) Scientific research and training (23) Immunization planning (24) Health education (25) Others | If the “(1)Administrative department” was chosen, the variable ”Administrative department” was labeled as ”Yes”.  No = 0; Yes = 1; |
| B2 | Operational department | The description was same as B1. | If any choice except “(1)Administrative department” was chosen, the variable ”Operational department” was labeled as ”Yes”.  No = 0; Yes = 1; |
| B3 | Work year | Work year: ____ years | ≤ 5 = 1; 6-10 = 2; 11-20 = 3; ≥ 21 = 4; |
| B4 | Workload | How do you feel about your current work intensity? (1) Very low (2) Low (3) Moderate (4) High (5) Very high | A score of 3 or below indicated “Low”, while a score above 3 indicated “High”.  Low = 0; High = 1; |
| B5 | Work fatigue | How often do you feel work fatigue during work? (1) Never (2) Rarely (3) Occasionally (4) Often (5) Almost every day | A score of 3 or below indicated “Low”, while a score above 3 indicated “High”.  Low = 0; High = 1; |
| D1 | College education satisfaction | Are you satisfied with the current college education for public health personnel? | Likert 5, from 1 (hardly satisfied) to 5 (strongly satisfied).  Low = 0; High = 1; |
| D2 | Continuing education satisfaction | Are you satisfied with the current continuing education for public health personnel? | Likert 5, from 1 (hardly satisfied) to 5 (strongly satisfied).  Low = 0; High = 1; |
| E1 | Pre-job training satisfaction | Are you satisfied with the current pre-job training for public health personnel? | Likert 5, from 1 (hardly satisfied) to 5 (strongly satisfied).  Low = 0; High = 1; |
| E2 | On-job training satisfaction | Are you satisfied with the current on-job training for public health personnel? | Likert 5, from 1 (hardly satisfied) to 5 (strongly satisfied).  Low = 0; High = 1; |
| F1 | Self-improvement needs | What reasons would prompt you to participate in the training? [multiple choices]   1. Self-improvement needs (2) Job-promotion needs (3) Obtain educational credits (4) Salary-improvement needs (5) Social needs: Expand the circle of work communication (6) Job hopping needs (7) Broaden one's horizons (8) Study abroad (9) Others | If the choices were chosen, the corresponding variables were marked as “High”; otherwise, they were marked as “Low”.  Low = 0; High = 1; |
| F2 | Job-promotion needs |  |  |
| F3 | Salary-improvement needs |  |  |
| F4 | Social needs |  |  |
| F5 | Job hopping needs |  |  |
| G1 | Job achievement | Please rate the following statements on a scale of 0 to 10 based on your own situation. The higher the score, the more in line with the description.   1. Job achievement (2) Team cohesion (3) Job identify (4) Work enthusiasm (5) Self support for job | The score below 8 was marked as “Low”, while the score was 8 or above 8 was marked as “High”.  Low = 0; High = 1; |
| G2 | Team cohesion |  |  |
| G3 | Job identify |  |  |
| G4 | Work enthusiasm |  |  |
| G5 | Income satisfaction | Are you satisfied with your monthly income? | Likert 5, from 1 (hardly satisfied) to 5 (strongly satisfied).  Low = 0; High = 1; |
| H1 | Self support for job | The description was the same as G1-G4. | The score below 8 was marked as “Low”, while the score was 8 or above 8 was marked as “High”.  Low = 0; High = 1; |
| H2 | Family support for job | How much support does your family offer for your work? | Likert 5. The score below median was marked as “Low”, otherwise, it was “High”.  Low = 0; High = 1; |
| H3 | Social acceptance for job | How do you think the current job you are engaged in is recognized by society? | Likert 5. The score below median was marked as “Low”, otherwise, it was “High”.  Low = 0; High = 1; |
| I1 | Inadequate funding for public health education | What are the key challenges in public health personnel development? [multiple choices]  “I” indicated from the perspective of funds support;  “J” indicated from the perspective of training weakness;  “K” indicated from the perspective of trainees’ subjective issues. | If the choices were chosen, the corresponding variables were marked as “Yes”; otherwise, they were marked as “No”.  No = 0; Yes = 1; |
| I2 | Inadequate organizational funds for training |  |  |
| J1 | Insufficient support from the organization for training |  |  |
| J2 | Disconnect between public health education and work |  |  |
| J3 | Training content cannot be set as needed |  |  |
| J4 | Training content is not highly relevant to the position |  |  |
| J5 | Lack of practical skills training |  |  |
| J6 | Lack of a systematic training assessment system |  |  |
| J7 | Lack of advanced curriculum and teaching methods |  |  |
| J8 | Lack of advanced teaching and scientific research experimental equipment |  |  |
| J9 | Lack of standardized training base |  |  |
| J10 | Insufficient informationization construction |  |  |
| J11 | Unreasonable structure of teaching staff |  |  |
| J12 | The trainers lack integrated and systematic strategic thinking |  |  |
| J13 | Training is just a formality |  |  |
| K1 | No work challenges, no learning required |  |  |
| K2 | Lack of common goals between individuals and units |  |  |
| K3 | Short training time and didn’t get it |  |  |
| K4 | Efforts and rewards are not proportional |  |  |
| K5 | Multiple responsibilities in one position, no time to study |  |  |
| K6 | Conflict of work time and study time |  |  |
| K7 | Lack initiative training for trainees |  |  |

Table S2. Fitting index of the latent class analysis.

| Model | AIC | BIC | SSA-BIC | Entropy | LMR-LRT p-value | BLRT  p-value | Class proportion |
| --- | --- | --- | --- | --- | --- | --- | --- |
|  |  |  |  |  |  |  |  |
| 1 | 383263.201 | 383440.448 | 383364.179 | N/A | N/A | N/A | 1 |
| 2 | 283344.424 | 283706.303 | 283550.587 | 0.953 | 0.333 | <0.0001 | 0.52/0.48 |
| 3 | 257285.952 | 257832.464 | 257597.301 | 0.938 | <0.0001 | <0.0001 | 0.41/0.24/0.35 |
| 4 | 240307.619 | 241038.764 | 240724.153 | 0.939 | <0.0001 | <0.0001 | 0.25/0.15/0.25/0.35 |
| 5 | 232924.211 | 233839.988 | 233445.93 | 0.927 | <0.0001 | <0.0001 | 0.20/0.24/0.14/0.19/0.22 |
| 6 | 228065.640 | 229166.050 | 228692.545 | 0.922 | <0.0001 | <0.0001 | 0.09/0.17/0.21/0.11/0.18/0.24 |

Note: AIC, Akaike information criteria; BIC, Bayesian information criterion; SSA-BIC, sample-size adjusted Bayesian information criterion; LMRT, Lo–Mendell-Rubin Likelihood Ratio Test; BLRT, Bootstrapped likelihood ratio test; N/A, not applicable.

Table S3. The estimated item probabilities of the optimal latent class analysis.

| Item |  | Class 1 (25.3%) | Class 2 (15.1%) | Class 3 (24.7%) | Class 4 (34.9%) |
| --- | --- | --- | --- | --- | --- |
| Item 1 | Public Health Service Capability | 0.108 | 0.809 | 0.501 | 0.949 |
| Item 2 | Laboratory Testing and Detection Capability | 0.147 | 0.67 | 0.317 | 0.822 |
| Item 3 | Epidemiological Tracing Capability | 0.113 | 0.881 | 0.429 | 0.963 |
| Item 4 | Surveillance and Early Warning Capability | 0.067 | 0.879 | 0.351 | 0.964 |
| Item 5 | Risk Identification and Assessment Capability | 0.027 | 0.841 | 0.313 | 0.975 |
| Item 6 | Emergency Response and Management Capability | 0.08 | 0.95 | 0.484 | 0.994 |
| Item 7 | Field Investigation and Control Capability | 0.071 | 0.942 | 0.488 | 0.996 |
| Item 8 | Community Diagnosis and Care Capability | 0.021 | 0.707 | 0.131 | 0.892 |
| Item 9 | Chronic Disease Control and Management Capability | 0.049 | 0.772 | 0.217 | 0.944 |
| Item 10 | Health Education and Promotion Capability | 0.064 | 0.817 | 0.341 | 0.972 |
| Item 11 | Basic Clinical Skills | 0.052 | 0.671 | 0.17 | 0.858 |
| Item 12 | Document Writing Ability | 0.028 | 0.112 | 0.216 | 0.627 |
| Item 13 | Scientific Research Capability | 0.067 | 0.226 | 0.447 | 0.791 |
| Item 14 | Data Analysis Capability | 0.063 | 0.253 | 0.434 | 0.813 |
| Item 15 | Learning Capability | 0.194 | 0.519 | 0.75 | 0.941 |
| Item 16 | Innovation Capability | 0.046 | 0.203 | 0.409 | 0.81 |
| Item 17 | Policy Interpretation and Implementation Capability | 0.11 | 0.424 | 0.717 | 0.936 |
| Item 18 | Leadership Decision Making Capability | 0.027 | 0.093 | 0.628 | 0.919 |
| Item 19 | Organizational Coordination Ability | 0.042 | 0.189 | 0.839 | 0.979 |
| Item 20 | Publicity and Mobilization Ability | 0.033 | 0.202 | 0.8 | 0.982 |
| Item 21 | Public Opinion Management Capability | 0.016 | 0.127 | 0.654 | 0.965 |
| Item 22 | Team Collaboration Ability | 0.246 | 0.619 | 0.965 | 0.997 |
| Item 23 | Motivation Capability | 0.107 | 0.399 | 0.813 | 0.98 |
| Item 24 | Resilience Capability | 0.174 | 0.463 | 0.824 | 0.964 |

Table S4. Characteristics by training needs among public health personnel.

| Variables | | Total (%) | Low Needs (%) | High Needs (%) | Χ2 | P-value |
| --- | --- | --- | --- | --- | --- | --- |
|  |  | N = 11912 | n = 5927 (49.8) | n = 5985 (50.2) |  |  |
| Sex (A1) | | | | | 5.047 | .025 |
|  | Male | 3876 (32.5) | 1986 (33.5) | 1890 (31.6) |  |  |
|  | Female | 8036 (67.5) | 3941 (66.5) | 4095 (68.4) |  |  |
| Age (A2) | | | | | 54.005 | ＜ .001 |
|  | ≤ 30 | 3208 (26.9) | 1489 (25.1) | 1719 (28.7) |  |  |
|  | 31-40 | 3332 (28.0) | 1566 (26.4) | 1766 (29.5) |  |  |
|  | 41-50 | 3010 (25.3) | 1606 (27.1) | 1404 (23.5) |  |  |
|  | ≥ 51 | 2362 (19.8) | 1266 (21.4) | 1096 (18.3) |  |  |
| Educational level (A3) | | | | | 69.555 | ＜ .001 |
|  | College degree or below | 3472 (29.1) | 1914 (32.3) | 1558 (26.0) |  |  |
|  | Bachelor's degree | 7400 (62.1) | 3573 (60.3) | 3827 (63.9) |  |  |
|  | Above bachelor's degree | 1040 (8.7) | 440 (7.4) | 600 (10.0) |  |  |
| Nationally-certified Professional Title (A4) | | | | | 4.121 | .13 |
|  | Junior | 6710 (56.3) | 3344 (56.4) | 3366 (56.2) |  |  |
|  | Intermediate | 2272 (19.1) | 1092 (18.4) | 1180 (19.7) |  |  |
|  | Senior | 2930 (24.6) | 1491 (25.2) | 1439 (24.0) |  |  |
| Region (A5) | | | | | 131.156 | ＜ .001 |
|  | Heilongjiang Province | 2289 (19.2) | 1203 (20.3) | 1086 (18.1) |  |  |
|  | Jilin Province | 3454 (29.0) | 1945 (32.8) | 1509 (25.2) |  |  |
|  | Liaoning Province | 1559 (13.1) | 756 (12.8) | 803 (13.4) |  |  |
|  | Inner Mongolia Autonomous | 4610 (38.7) | 2023 (34.1) | 2587 (43.2) |  |  |
| Institutional classification (A6) | | | | | 13.432 | .001 |
|  | Provincial and municipal level | 708 (5.9) | 329 (5.6) | 379 (6.3) |  |  |
|  | Prefectural level | 3351 (28.1) | 1597 (26.9) | 1754 (29.3) |  |  |
|  | County/district level | 7853 (65.9) | 4001 (67.5) | 3852 (64.4) |  |  |
| Income monthly (RMB) (A7) | | | | | 5.965 | .20 |
|  | ≤ 3000 | 1603 (13.5) | 834 (14.1) | 769 (12.8) |  |  |
|  | 3001-5000 | 6138 (51.5) | 3037 (51.2) | 3101 (51.8) |  |  |
|  | 5001-7000 | 2872 (24.1) | 1410 (23.8) | 1462 (24.4) |  |  |
|  | 7001-8000 | 725 (6.1) | 348 (5.9) | 377 (6.3) |  |  |
|  | ≥ 8001 | 574 (4.8) | 298 (5.0) | 276 (4.6) |  |  |
| Administrative department (B1) | | | | | 1.229 | .27 |
|  | No | 9909 (83.2) | 4953 (83.6) | 4956 (82.8) |  |  |
|  | Yes | 2003 (16.8) | 974 (16.4) | 1029 (17.2) |  |  |
| Operational department (B2) | | | | | 1.008 | .32 |
|  | No | 3191 (26.8) | 1612 (27.2) | 1579 (26.4) |  |  |
|  | Yes | 8721 (73.2) | 4315 (72.8) | 4406 (73.6) |  |  |
| Work years (B3) | | | | | 60.987 | ＜ .001 |
|  | ≤ 5 | 3844 (32.3) | 1775 (29.9) | 2069 (34.6) |  |  |
|  | 6-10 | 1532 (12.9) | 743 (12.5) | 789 (13.2) |  |  |
|  | 11-20 | 2152 (18.1) | 1027 (17.3) | 1125 (18.8) |  |  |
|  | ≥ 21 | 4384 (36.8) | 2382 (40.2) | 2002 (33.5) |  |  |
| Workload (B4) | | | | | 43.155 | ＜ .001 |
|  | Low | 6782 (56.9) | 3552 (59.9) | 3230 (54.0) |  |  |
|  | High | 5130 (43.1) | 2375 (40.1) | 2755 (46.0) |  |  |
| Work fatigue (B5) | | | | | 1.131 | .29 |
|  | Low | 6929 (58.2) | 3419 (57.7) | 3510 (58.6) |  |  |
|  | High | 4983 (41.8) | 2508 (42.3) | 2475 (41.4) |  |  |
| Competency pattern (C) | | | | | 216.549 | ＜ .001 |
|  | Novice | 3008 (25.3) | 1799 (30.4) | 1209 (20.2) |  |  |
|  | Public health specialist | 1798 (15.1) | 945 (15.9) | 853 (14.3) |  |  |
|  | Management talent | 2944 (24.7) | 1410 (23.8) | 1534 (25.6) |  |  |
|  | Versatile talent | 4162 (34.9) | 1773 (29.9) | 2389 (39.9) |  |  |
| College education satisfaction (D1) | | | | | 385.323 | ＜ .001 |
|  | Low | 6403 (53.8) | 3720 (62.8) | 2683 (44.8) |  |  |
|  | High | 5509 (46.2) | 2207 (37.2) | 3302 (55.2) |  |  |
| Continuing education satisfaction (D2) | | | | | 1.342 | .25 |
|  | Low | 7570 (63.5) | 3797 (64.1) | 3773 (63.0) |  |  |
|  | High | 4342 (36.5) | 2130 (35.9) | 2212 (37.0) |  |  |
| Pre-job training satisfaction (E1) | | | | | 0.510 | .48 |
|  | Low | 2405 (20.2) | 1181 (19.9) | 1224 (20.5) |  |  |
|  | High | 9507 (79.8) | 4746 (80.1) | 4761 (79.5) |  |  |
| On-job training satisfaction (E2) | | | | | 999.917 | ＜ .001 |
|  | Low | 6711 (56.3) | 4195 (70.8) | 2516 (42.0) |  |  |
|  | High | 5201 (43.7) | 1732 (29.2) | 3469 (58.0) |  |  |
| Self-improvement needs (F1) | | | | | 292.096 | ＜ .001 |
|  | Low | 1648 (13.8) | 1142 (19.3) | 506 (8.5) |  |  |
|  | High | 10264 (86.2) | 4785 (80.7) | 5479 (91.5) |  |  |
| Job-promotion needs (F2) | | | | | 35.406 | ＜ .001 |
|  | Low | 5411 (45.4) | 2854 (48.2) | 2557 (42.7) |  |  |
|  | High | 6501 (54.6) | 3073 (51.8) | 3428 (57.3) |  |  |
| Salary-improvement needs (F3) | | | | | 0.743 | .39 |
|  | Low | 6886 (57.8) | 3403 (57.4) | 3483 (58.2) |  |  |
|  | High | 5026 (42.2) | 2524 (42.6) | 2502 (41.8) |  |  |
| Social needs (F4) | | | | | 0.284 | .59 |
|  | Low | 7903 (66.3) | 3946 (66.6) | 3957 (66.1) |  |  |
|  | High | 4009 (33.7) | 1981 (33.4) | 2028 (33.9) |  |  |
| Job hopping needs (F5) | | | | | 0.093 | .76 |
|  | Low | 9515 (79.9) | 4741 (80.0) | 4774 (79.8) |  |  |
|  | High | 2397 (20.1) | 1186 (20.0) | 1211 (20.2) |  |  |
| Job achievement (G1) | | | | | 117.702 | ＜ .001 |
|  | Low | 6122 (51.4) | 3342 (56.4) | 2780 (46.4) |  |  |
|  | High | 5790 (48.6) | 2585 (43.6) | 3205 (53.6) |  |  |
| Team cohesion (G2) | | | | | 194.751 | ＜ .001 |
|  | Low | 5194 (43.6) | 2962 (50.0) | 2232 (37.3) |  |  |
|  | High | 6718 (56.4) | 2965 (50.0) | 3753 (62.7) |  |  |
| Job identification (G3) | | | | | 150.459 | ＜ .001 |
|  | Low | 5374 (45.1) | 3007 (50.7) | 2367 (39.5) |  |  |
|  | High | 6538 (54.9) | 2920 (49.3) | 3618 (60.5) |  |  |
| Work enthusiasm (G4) | | | | | 0.030 | .86 |
|  | Low | 4770 (40.0) | 2378 (40.1) | 2392 (40.0) |  |  |
|  | High | 7142 (60.0) | 3549 (59.9) | 3593 (60.0) |  |  |
| Income satisfaction (G5) | | | | | 16.872 | ＜ .001 |
|  | Low | 6156 (51.7) | 2951 (49.8) | 3205 (53.6) |  |  |
|  | High | 5756 (48.3) | 2976 (50.2) | 2780 (46.4) |  |  |
| Self support for job (H1) | | | | | 1.195 | .27 |
|  | Low | 4853 (40.7) | 2444 (41.2) | 2409 (40.3) |  |  |
|  | High | 7059 (59.3) | 3483 (58.8) | 3576 (59.7) |  |  |
| Family support for job (H2) | | | | | 1.766 | .18 |
|  | Low | 2710 (22.8) | 1318 (22.2) | 1392 (23.3) |  |  |
|  | High | 9202 (77.2) | 4609 (77.8) | 4593 (76.7) |  |  |
| Social acceptance for job (H3) | | | | | 0.557 | .46 |
|  | Low | 6483 (54.4) | 3246 (54.8) | 3237 (54.1) |  |  |
|  | High | 5429 (45.6) | 2681 (45.2) | 2748 (45.9) |  |  |
| Inadequate funding for public health education (I1) | | | | | 1.127 | .29 |
|  | Low | 4224 (35.5) | 2074 (35.0) | 2150 (35.9) |  |  |
|  | High | 7688 (64.5) | 3853 (65.0) | 3835 (64.1) |  |  |
| Inadequate organizational funds for training (I2) | | | | | 9.843 | .002 |
|  | No | 6440 (54.1) | 3119 (52.6) | 3321 (55.5) |  |  |
|  | Yes | 5472 (45.9) | 2808 (47.4) | 2664 (44.5) |  |  |
| Insufficient support from the organization for training (J1) | | | | | 0.074 | .79 |
|  | No | 10167 (85.4) | 5064 (85.4) | 5103 (85.3) |  |  |
|  | Yes | 1745 (14.6) | 863 (14.6) | 882 (14.7) |  |  |
| Disconnect between public health education and work (J2) | | | | | 0.970 | .33 |
|  | No | 6712 (56.3) | 3313 (55.9) | 3399 (56.8) |  |  |
|  | Yes | 5200 (43.7) | 2614 (44.1) | 2586 (43.2) |  |  |
| Training content cannot be set as needed (J3) | | | | | 0.000 | .99 |
|  | No | 6977 (58.6) | 3471 (58.6) | 3506 (58.6) |  |  |
|  | Yes | 4935 (41.4) | 2456 (41.4) | 2479 (41.4) |  |  |
| Training content is not highly relevant to the position (J4) | | | | | 0.625 | .43 |
|  | No | 8260 (69.3) | 4090 (69.0) | 4170 (69.7) |  |  |
|  | Yes | 3652 (30.7) | 1837 (31.0) | 1815 (30.3) |  |  |
| Lack of practical skills training (J5) | | | | | 0.062 | .80 |
|  | No | 6901 (57.9) | 3427 (57.8) | 3474 (58.0) |  |  |
|  | Yes | 5011 (42.1) | 2500 (42.2) | 2511 (42.0) |  |  |
| Lack of a systematic training assessment system (J6) | | | | | 19.907 | ＜ .001 |
|  | No | 8037 (67.5) | 4113 (69.4) | 3924 (65.6) |  |  |
|  | Yes | 3875 (32.5) | 1814 (30.6) | 2061 (34.4) |  |  |
| Lack of advanced curriculum and teaching methods (J7) | | | | | 0.788 | .38 |
|  | No | 7788 (65.4) | 3852 (65.0) | 3936 (65.8) |  |  |
|  | Yes | 4124 (34.6) | 2075 (35.0) | 2049 (34.2) |  |  |
| Lack of advanced teaching and scientific research experimental equipment (J8) | | | | | 2.089 | .15 |
|  | No | 7859 (66.0) | 3873 (65.3) | 3986 (66.6) |  |  |
|  | Yes | 4053 (34.0) | 2054 (34.7) | 1999 (33.4) |  |  |
| Lack of standardized training base (J9) | | | | | 1.089 | .30 |
|  | No | 6954 (58.4) | 3432 (57.9) | 3522 (58.8) |  |  |
|  | Yes | 4958 (41.6) | 2495 (42.1) | 2463 (41.2) |  |  |
| Insufficient informationization construction (J10) | | | | | 1.750 | .19 |
|  | No | 8090 (67.9) | 4059 (68.5) | 4031 (67.4) |  |  |
|  | Yes | 3822 (32.1) | 1868 (31.5) | 1954 (32.6) |  |  |
| Unreasonable structure of trainers’ team (J11) | | | | | 0.110 | .74 |
|  | No | 8958 (75.2) | 4465 (75.3) | 4493 (75.1) |  |  |
|  | Yes | 2954 (24.8) | 1462 (24.7) | 1492 (24.9) |  |  |
| The trainers lack integrated and systematic strategic thinking (J12) | | | | | 0.239 | .63 |
|  | No | 9644 (81.0) | 4809 (81.1) | 4835 (80.8) |  |  |
|  | Yes | 2268 (19.0) | 1118 (18.9) | 1150 (19.2) |  |  |
| Training is just a formality (J13) | | | | | 0.081 | .78 |
|  | No | 8114 (68.1) | 4030 (68.0) | 4084 (68.2) |  |  |
|  | Yes | 3798 (31.9) | 1897 (32.0) | 1901 (31.8) |  |  |
| No work challenges, no learning required (K1) | | | | | 0.724 | .40 |
|  | No | 9893 (83.1) | 4905 (82.8) | 4988 (83.3) |  |  |
|  | Yes | 2019 (16.9) | 1022 (17.2) | 997 (16.7) |  |  |
| Lack of common goals between individuals and units (K2) | | | | | 0.118 | .73 |
|  | No | 9193 (77.2) | 4582 (77.3) | 4611 (77.0) |  |  |
|  | Yes | 2719 (22.8) | 1345 (22.7) | 1374 (23.0) |  |  |
| Short training time and trainees didn’t get it (K3) | | | | | 0.830 | .36 |
|  | No | 8216 (69.0) | 4111 (69.4) | 4105 (68.6) |  |  |
|  | Yes | 3696 (31.0) | 1816 (30.6) | 1880 (31.4) |  |  |
| Efforts and rewards are not proportional (K4) | | | | | 1.226 | .27 |
|  | No | 8432 (70.8) | 4168 (70.3) | 4264 (71.2) |  |  |
|  | Yes | 3480 (29.2) | 1759 (29.7) | 1721 (28.8) |  |  |
| Multiple responsibilities in one position, no time to study (K5) | | | | | 2.533 | .11 |
|  | No | 6460 (54.2) | 3171 (53.5) | 3289 (55.0) |  |  |
|  | Yes | 5452 (45.8) | 2756 (46.5) | 2696 (45.0) |  |  |
| Conflict of work time and study time (K6) | | | | | 9.244 | .002 |
|  | No | 6130 (51.5) | 3133 (52.9) | 2997 (50.1) |  |  |
|  | Yes | 5782 (48.5) | 2794 (47.1) | 2988 (49.9) |  |  |
| Lack initiative training for trainees (K7) | | | | | 1.779 | .18 |
|  | No | 9372 (78.7) | 4693 (79.2) | 4679 (78.2) |  |  |
|  | Yes | 2540 (21.3) | 1234 (20.8) | 1306 (21.8) |  |  |

Table S5. Performance metrics of the four machine learning models in the train set and test set.

| Data | Models | Accuracy (95% CI) | Precision (95% CI) | Recall (95% CI) | F1 score (95% CI) | AUC (95% CI) |
| --- | --- | --- | --- | --- | --- | --- |
| Train set | LR | 0.6532 | 0.6487 | 0.6757 | 0.6619 | 0.699 |
|  | RF | 0.5086 | 0.51 | 0.5592 | 0.5335 | 0.709 |
|  | LASSO | 0.6483 | 0.6775 | 0.5726 | 0.6206 | 0.697 |
|  | XGBoost | 0.656 | 0.6604 | 0.6492 | 0.6547 | 0.706 |
| Test set | LR | 0.6488 (0.6328, 0.6655) | 0.6464 (0.6231, 0.6694) | 0.6641 (0.6414, 0.6862) | 0.6551 (0.6366, 0.6739) | 0.698 (0.6808, 0.7153) |
|  | RF | 0.5035 (0.4848, 0.5169) | 0.5054 (0.4791, 0.5245) | 0.5454 (0.527, 0.5581) | 0.5247 (0.503, 0.539) | 0.688 (0.6705, 0.7049) |
|  | LASSO | 0.6429 (0.6272, 0.6588) | 0.6715 (0.6461, 0.6971) | 0.5660 (0.5422, 0.5870) | 0.6143 (0.5937, 0.6329) | 0.692 (0.6747, 0.7088) |
|  | XGBoost | 0.6485 (0.6328, 0.6653) | 0.6564 (0.6314, 0.6820) | 0.6301 (0.6056, 0.6512) | 0.6430 (0.6248, 0.6611) | 0.702 (0.6847, 0.7192) |

Note: CI, confidence interval.

Table S6. AUC of four machine learning models grouped by sex, age, education level, and job title.

|  |  | **LR** | **RF** | **LASSO** | **XGBoost** |
| --- | --- | --- | --- | --- | --- |
| **Sex** | Male | 0.718 | 0.720 | 0.716 | 0.723 |
|  | Female | 0.698 | 0.694 | 0.687 | 0.696 |
| **Age** | ≤ 30 | 0.705 | 0.713 | 0.704 | 0.714 |
|  | 31-40 | 0.697 | 0.702 | 0.693 | 0.705 |
|  | 41-50 | 0.697 | 0.692 | 0.693 | 0.700 |
|  | ≥ 51 | 0.690 | 0.698 | 0.691 | 0.691 |
| **Educational level** | College degree or below | 0.726 | 0.731 | 0.727 | 0.731 |
|  | Bachelor's degree | 0.694 | 0.699 | 0.691 | 0.700 |
|  | Above bachelor's degree | 0.649 | 0.651 | 0.644 | 0.655 |
| **Nationally-certified Professional Title (Job title)** | Junior | 0.714 | 0.720 | 0.712 | 0.722 |
|  | Intermediate | 0.685 | 0.686 | 0.681 | 0.690 |
|  | Senior | 0.674 | 0.675 | 0.671 | 0.676 |

Figure S1. The average score of the items in the four different competency patterns.

Figure S2. Distribution of competency patterns across demographic and job-related characteristics.


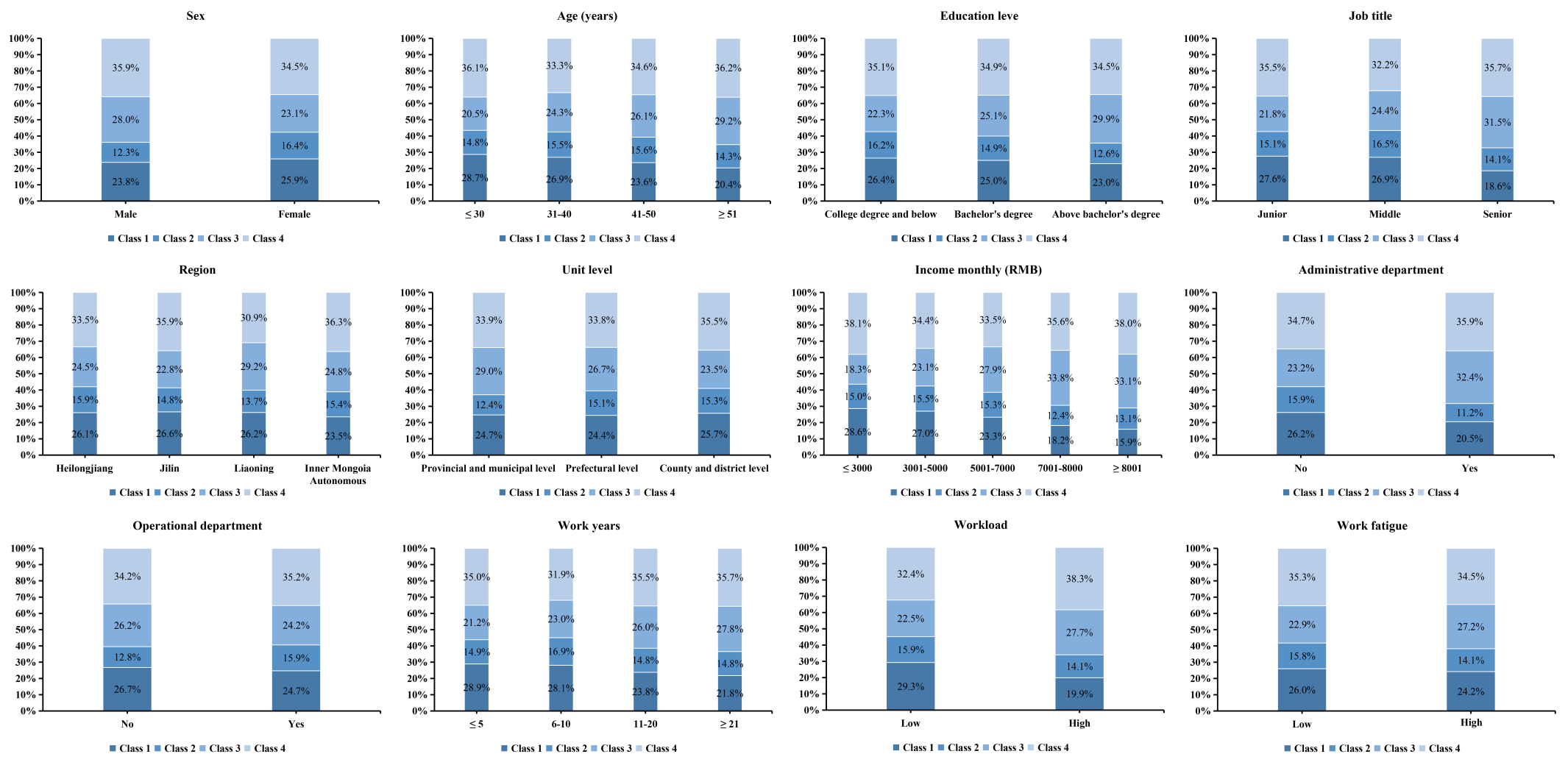


Figure S3. The confusion matrices of the four machine learning models in the test set.


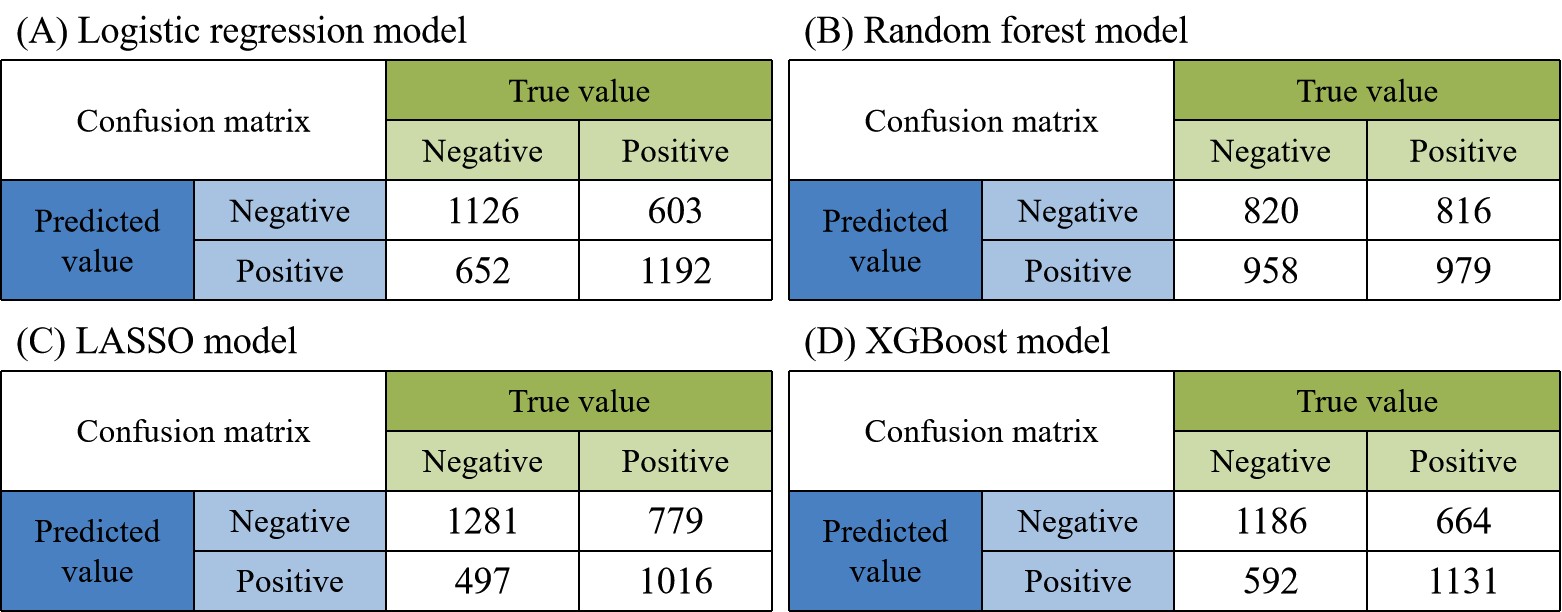

Supplement: Supplementary file 1 — Supplementary Material 1 [file 12889_2025_23393_MOESM1_ESM.docx]
